# Physiology of deep closed circuit rebreather mixed gas diving: vascular gas emboli and biological changes during a week-long liveaboard safari

**DOI:** 10.3389/fphys.2024.1395846

**Published:** 2024-04-10

**Authors:** Costantino Balestra, Clément Lévêque, Simona Mrakic-Sposta, Alessandra Vezzoli, Pierre Wauthy, Peter Germonpré, Frauke Tillmans, François Guerrero, Pierre Lafère

**Affiliations:** ^1^ Environmental, Occupational, Aging (Integrative) Physiology Laboratory, Haute Ecole Bruxelles-Brabant (HE2B), Brussels, Belgium; ^2^ Physical Activity Teaching Unit, Motor Sciences Department, Université Libre de Bruxelles (ULB), Brussels, Belgium; ^3^ DAN Europe Research Division (Roseto-Brussels), Brussels, Belgium; ^4^ Institute of Clinical Physiology, National Research Council (CNR), Milan, Italy; ^5^ Department of Cardiac Surgery, CHU Brugmann, Université Libre de Bruxelles (ULB), Brussels, Belgium; ^6^ Centre for Hyperbaric Oxygen Therapy, Queen Astrid Military Hospital, Brussels, Belgium; ^7^ Divers Alert Network, Durham, NC, United States; ^8^ Laboratoire ORPHY EA 4324, University Brest, Brest, France

**Keywords:** acute phase, decompression physiology, hormesis, human, oxyinflammation, reactive oxygen species (ROS), vascular gas emboli (VGE)

## Abstract

**Introduction:** Diving decompression theory hypothesizes inflammatory processes as a source of micronuclei which could increase related risks. Therefore, we tested 10 healthy, male divers. They performed 6–8 dives with a maximum of two dives per day at depths ranging from 21 to 122 msw with CCR mixed gas diving.

**Methods:** Post-dive VGE were counted by echocardiography. Saliva and urine samples were taken before and after each dive to evaluate inflammation: ROS production, lipid peroxidation (8-iso-PGF2), DNA damage (8-OH-dG), cytokines (TNF-α, IL-6, and neopterin).

**Results:** VGE exhibits a progressive reduction followed by an increase (*p* < 0.0001) which parallels inflammation responses. Indeed, ROS, 8-iso-PGF2, IL-6 and neopterin increases from 0.19 ± 0.02 to 1.13 ± 0.09 μmol.min^−1^ (*p* < 0.001); 199.8 ± 55.9 to 632.7 ± 73.3 ng.mg^−1^ creatinine (*p* < 0.0001); 2.35 ± 0.54 to 19.5 ± 2.96 pg.mL^−1^ (*p* < 0.001); and 93.7 ± 11.2 to 299 ± 25.9 μmol·mol^−1^ creatinine (*p* = 0.005), respectively. The variation after each dive was held constant around 158.3% ± 6.9% (*p* = 0.021); 151.4% ± 5.7% (*p* < 0.0001); 176.3% ± 11.9% (*p* < 0.0001); and 160.1% ± 5.6% (*p* < 0.001), respectively.

**Discussion:** When oxy-inflammation reaches a certain level, it exceeds hormetic coping mechanisms allowing second-generation micronuclei substantiated by an increase of VGE after an initial continuous decrease consistent with a depletion of “first generation” pre-existing micronuclei.

## 1 Introduction

Although SCUBA (self-contained underwater breathing apparatus) diving procedures have become safer over time, decompression sickness (DCS) remains a risk, that can be life-threatening. Its pathophysiology is traditionally viewed as related to gas bubble formation during and following ascent, due to ambient pressure drop (Papadopoulou et al., 2014). Then, the primary mechanisms of bubble production is related to the number and size of preexisting endothelial gas micronuclei also called static metabolic bubbles (SMB) (Imbert et al., 2019). However, the inconsistent presence of bubbles in human studies indicates the need for more dedicated research on their generation and their evolution over several consecutive days (Dugrenot et al., 2021; Balestra et al., 2022b; Gouin et al., 2023). More, the exact pathophysiological mechanisms linking Vascular Gas Emboli (VGE) to DCS are still unclear. Therefore, additional pathological mechanisms contributing to the occurrence of DCS might be involved (Vann et al., 2011; Møllerløkken et al., 2012). For example, functional changes in vascular wall as shown by impaired flow-mediated dilation (FMD), endothelial microparticles or oxidative stress following the dive have been linked to the presence of VGE (Brubakk et al., 2005; Obad et al., 2007; Thom et al., 2011; Lambrechts et al., 2013) but also to something called “decompression stress” without significant VGE presence, involving the direct effect of hydrostatic pressure or oxy-inflammation (Thom et al., 2015; Balestra et al., 2022a; Vezzoli et al., 2024). Nonetheless, the exact involvement of each of those mechanisms contributing to DCS remains unclear. It is however generally accepted that the absence of detectable post-dive VGE is correlated with a very low probability of DCS (Møllerløkken et al., 2012; Doolette, 2016).

**FIGURE 1:**
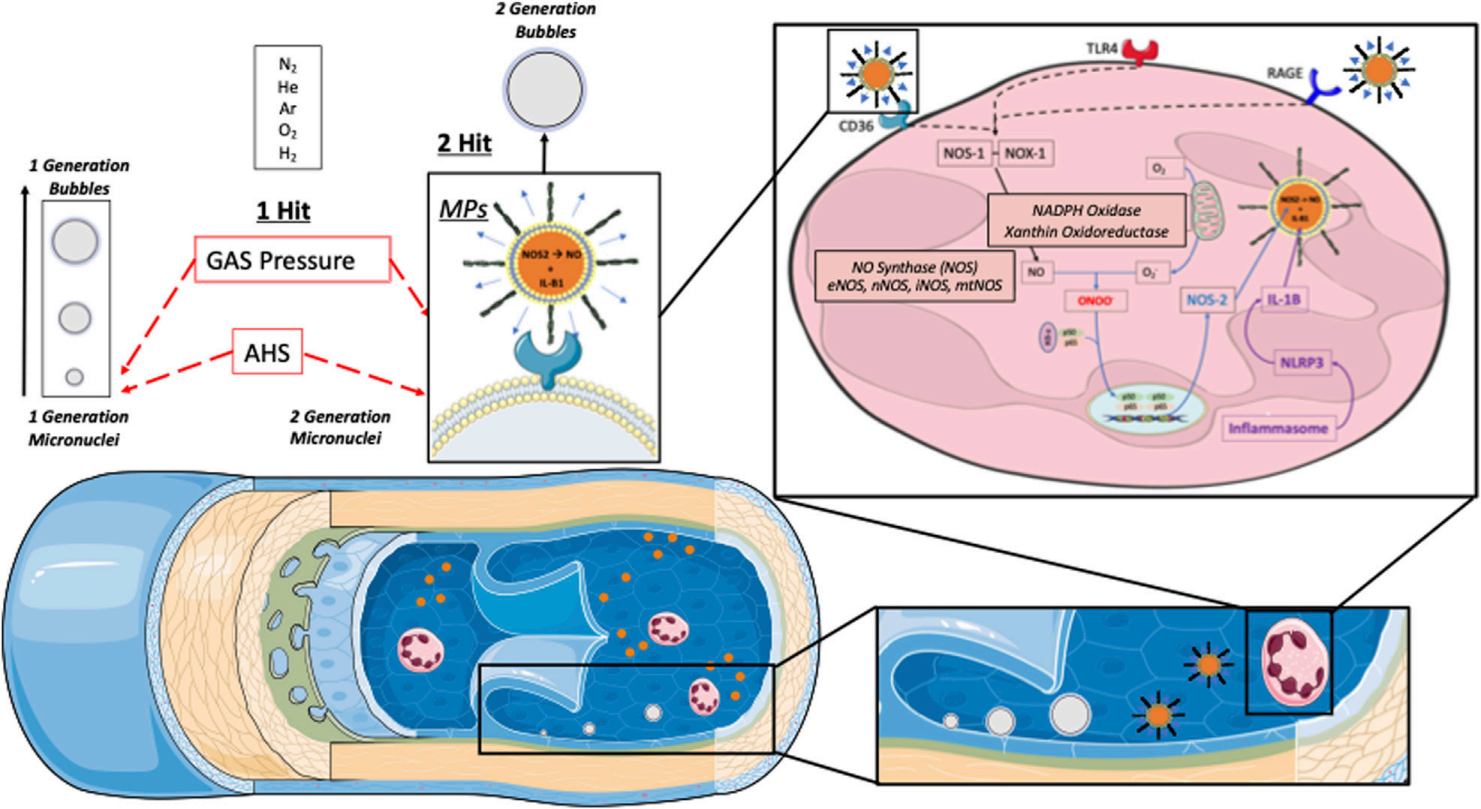
Schematic representation of actual Bubbles development theories during decompression phases. In supersaturated tissues and plasma, pre-existing “seeds” are needed to develop gas phase circulating vascular gas emboli (VGE). Those pre-existing micro-nuclei and other micronucleation sites [Active hydrophobic spots (AHS)] are considered “first generation micronuclei”. Once in circulation they can either be eliminated by the lungs (bubbles “filter”) or keep on growing while being fed by surrounding gas supersaturation. Another theory of Bubbles development is related to the Inflammatory response due to Oxygen partial pressure variation (Oxy-inflammation) or white blood cells elicited microparticles (MPs). Those MPs can contain gas and expand their volume as other micronuclei. They can also strip out some debris of the AHS which can also act as micronuclei. Another parameter to consider is the flow velocity that can be reduced in terminal vessels where even very small gas emboli can increase their volume and provoke ischemia since the longer time the longer “feeding” opportunity to increase volume and trigger some terminal vessel embolization that can fit some symptoms like for instance cerebellar or inner ear decompression diseases (Arieli, 2019).

These discrepancies have also prompted investigations focused on inflammatory pathways (Francis et al., 1988; Martin and Thom, 2002; Bigley et al., 2008; Mitchell et al., 2022). Also, our previous data after 1 week of diving using closed circuit rebreather (CCR) apparatus (Balestra et al., 2022b; Arya et al., 2023; Gouin et al., 2023) mandated investigating the inflammatory bubble production hypothesis (see Figure 1). This latter hypothesis is based on a “first and second hit” mechanism that cannot be considered fully subordinate to gas supersaturation. Indeed, this second mechanism is thought to be related to a pro-inflammatory/oxidative response following diving, probably mediated by the presence/generation of microparticles (MPs) (Arya et al., 2023). These MPs which are increased by repetitive and deep dives, may enlarge on decompression as any of micronuclei. More, actual data suggest that some of them possess enzyme activity, which provides a nucleation site for bubble formation (second generation micronuclei) (Thom et al., 2018; Bhopale et al., 2021; Arya et al., 2023). Because MPs are more numerous and possess higher NOS2 concentrations post-dive, somewhat in proportion to dive repetition and probably depth. This implies a greater potential for bubble nucleation and greater risk for bubble-induced vascular damage after such dives (Bhopale et al., 2021). It should be noted that, given the micro-dimensions of MPs, these “second-generation” bubbles related to MPs would still be below the detection limits of current ultrasound technology and may go beyond the pulmonary filter. Nevertheless, this mechanism could be at the root of some adverse effects of repetitive deep diving since these non-visible bubbles can be fed by inert gas accumulation and form measurable VGE (Vascular Gas Emboli) (Arya et al., 2023).

The inflammatory response is of particular interest in the setting of deep CCR technical open water diving due to oxygen partial pressure variation, as shown by the increase of IL-1β and NOS2 during repetitive deep CCR dives recently reported (Bhopale et al., 2021; Arya et al., 2023). Indeed, CCR diving differs from typical SCUBA diving because exhaled gas is recycled (“re-breathed”) after carbon dioxide removal and oxygen supplementation. Although CCR diving is often done with custom-mixed gases adapted to the time and depth planned, rather than air (see Table 1), to decrease the risks of oxygen toxicity and nitrogen narcosis, oxygen partial pressure is kept within narrow limits using oxygen sensors around 1.3 ATA. Therefore, it must be reminded that we recently demonstrated different cellular reactions and microparticles production in humans exposed to different levels of oxygen partial pressure (Balestra et al., 2022a). Interestingly, a partial pressure of 1.4 ATA was shown to influence MP production levels (Balestra et al., 2022a; Balestra et al., 2023a; Balestra et al., 2023b).

**TABLE 1:** Subjects’ characteristics before the first dive.

	Diver 1	Diver 2	Diver 3	Diver 4	Diver 5	Diver 6	Diver 7	Diver 8	Diver 9	Diver 10	Mean [range]
Age	58	27	44	54	33	50	43	54	46	35	44 [27–58]
Weight (Kg)	66	76.5	81	72.9	76	83.2	79	75.3	91	68	77 [66–91]
Height (cm)	180	183	173	181	193	181	172	178	175	178	179 [172–193]
BMI (Kg/m^2^)	20.4	22.8	27.1	22.2	20.4	25.4	26.7	23.8	29.7	21.5	24 [20.4–29.7]
BF (%)	15.0	18.9	25.5	16.2	18.3	24.9	24.0	17.8	23.3	14.6	19.9 [14.6–25.5]
Experience (years)	13	13	29	40	10	36	21	19	26	16	22 [10–40]
Experience (dives)	800	1,000	10,000	1,500	600	5,480	1800	700	700	7,000	2,958 [600–10,000]
Max depth (msw)	100	116	124	120	131	136	125	100	100	116	117 [100–136]

BMI, Body Mass Index; BF, Body Fat.

Since the use of CCR with a “hypoxic” breathing mixture allows the diver to reach much deeper depths than traditional open circuit divers, we wanted to consolidate our data on bubble evaluation after deep dives (Over 100 m depth). Therefore, we wanted to investigate how several days of intensive deep diving interfere with bubble production as well as oxidative and inflammatory markers. To the best of our knowledge, such a set of measurements in a group of divers has never been performed and follows some of our previous works. This description of VGE evolution through the week, in parallel with previously published inflammatory reactions (Arya et al., 2023) may add to the understanding of diving physiopathology.

## 2 Materials and methods

This is an observational field study conducted in accordance with the Declaration of Helsinki (World Medical Association, 2013), and approved by the Academic Ethical Committee of Brussels (B200-2020-088).

### 2.1 Population

After obtaining full written informed consent, 10 non-smoking, experienced divers (all males; mean age 44 ± 10 years; body mass index 24 ± 3.1 kg/m^2^, minimum certification “Hypoxic Trimix CCR diving” according to European norm EN 14153-2 or ISO 24801-2 with at least 300 logged dives) were recruited for this study (see Table 1).

None of the participants had a history of previous of DCS. All of them were in overall good health with regular but not excessive physical activity (aerobic exercise one to three times a week). No severe cardiac abnormalities were detected during the pre-inclusion medical assessment. However, some of them were under medication such as statins (n = 2), antihypertensive medication (n = 2), and sulfonylureas for blood sugar control (n = 2). Nonetheless, all participants held a valid medical clearance for diving at the time of the study.

Finally, they were instructed to abstain from any physical activity and diving for 72 h (Obad et al., 2007) prior to the experimental protocol. Alcohol intake was prohibited before diving, with a minimum pre-dive limit set 10 h before the next immersion (12.1 ± 0.3 h).

### 2.2 The dives

Dives were conducted from a 36-m dive boat based out of Hurghada—Egypt over a 6-days period with two dives a day (surface interval of 3–4 h) on day 1, and 2, followed by 4 days with a single dive at increasing depth up to 100 msw deep or beyond (Table 2). Weather conditions were good with daily air temperature between 25°C and 30°C and surface water temperature between 24°C and 28°C.

**TABLE 2:** Summary of the week schedule (Day/dive, Dn.dn). Number of divers involved in each dive, Diluent Mixes composition using a helium/O2 analyzer (ATA PRO, Analox, United Kingdom), Gradient Factors (Low (GF-L) and High (GF-H)), depth and dive duration (Runtime). Results are presented as mean and [min-max].

	D1.d1	D1.d2	D2.d1	D2.d2	D3.d1	D4.d1	D5.d1	D6.d1
Divers (n)	7	9	8	7	8	9	8	9
Oxygen (%)	12 [6–22]	13 [6–15]	11 [6–15]	11 [6–15]	8 [6–12]	8 [6–13]	8 [6–13]	7 [6–8]
Helium (%)	63 [20–88]	63 [49–88]	71 [49–88]	77 [49–88]	74 [58–88]	76 [59–88]	77 [59–88]	75 [70–88]
GF-L	47 [45–55]	46 [30–55]	46 [30–55]	46 [30–55]	46 [30–55]	46 [30–55]	[27–58]	46 [30–55]
GF-H	84 [80–90]	81 [70–90]	81 [70–90]	81 [70–90]	81 [70–90]	81 [70–90]	81 [70–90]	81 [70–90]
Depth (msw)	68 [66–71]	47 [37–76]	55 [26–86]	26 [21–32]	85 [67–88]	89 [69–105]	90 [69–105]	111 [100–122]
Time (min)	129 [105–161]	71 [58–159]	87 [74–198]	46 [42–48]	135 [51–225]	103 [71–127]	111 [68–134]	196 [107–280]

All divers used JJ-CCRs rebreather (JJ-CCR ApS, Presto, Denmark) equipped with an integrated multi-gas decompression computer, Petrel 2 (Shearwater Research Inc., Richmond, BC, Canada) with the build-in ZHL-16C algorithm, to incorporate the readings from the CCR O_2_ cells to calculate decompression requirements. These calculations were made based on a trimix diluent (A mixture containing Oxygen, Helium and Nitrogen in various proportions) customized according to depth and a fixed O_2_ set point at depth and for the ascent at 1.3 ATA. During the decompression phase, O_2_ set point was manually increased when arriving at a depth of six msw to be between 1.5 and 1.6 ATA (i.e., breathing 100% O_2_).

**FIGURE 2:**
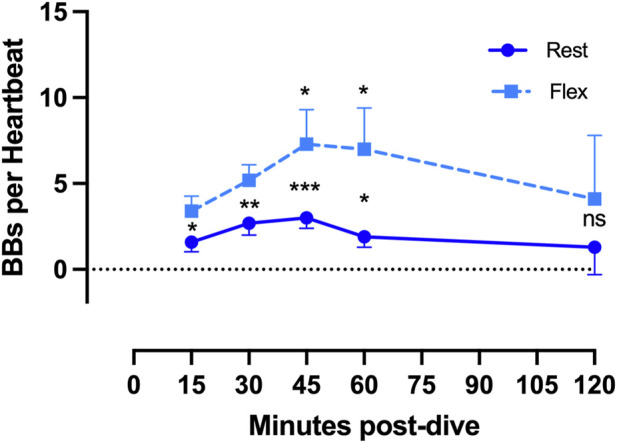
VGE production pattern based on the example of the last dive of the week (Maiden wreck, 101–122 msw). The two conditions (rest and flex) are for every time point 15, 30, 45, 60, 120 min after diving. (Data are presented as means ± SEM; n = 9; ns: not significant, *: *p* < 0.05, **: *p* < 0.01, ***: *p* < 0.001; Wilcoxon matched-pair rank signed test for intra-time comparison, Friedman test for inter-time comparison).

For safety reasons, divers also carried 3–4 off-board (“bail-out”) dive tanks to allow an independent return to the surface in the event of CCR failure. These “stage cylinders” (or “stages”) contained either trimix, nitrox or pure oxygen, depending on the decompression planning (Figure 2).

During the dive, divers used their diving computers to manage the decompression requirements. However, based on experience and utilization of dive-planning software, most participants had a fair idea of the required total dive time and final stop time for a given bottom time. Each diver had the liberty to personalize their own decompression schedule as desired, by selecting a different “conservatism” by means of gradient factors (GF), determining the maximum allowed supersaturation limits for the deep and shallow parts of the dive. In general, bottom time was planned based on estimated decompression obligations rather than gas requirements. It must be stressed out that it was not the purpose of the research team to recommend any specific diving procedure. Its only aim was to monitor divers and provide an insight into their post-dive physiological status and VGE dynamics. Therefore, the research team did not comment any of the diver’s choices even if most dives can be categorized as an “extreme exposure” according to the DCIEM decompression tables. Therefore, it was the decision of any diver to participate or not to each dive, which was not the case. These choices are resumed in Table 2.

### 2.3 Bubble analysis

Although imperfect, it is now accepted that research projects can use VGE data as a surrogate endpoint for decompression stress (Doolette, 2016; Balestra et al., 2019). According to current recommendations, cardiac echography is the gold standard for VGE detection (Møllerløkken et al., 2016). During field studies, bubbles are usually detected in the right atrium and ventricle.

Using two-dimensional echocardiography technique by two operators (CB, CL) and two echocardiography machines (M9, Mindray, Echomedic, Ghent, Belgium and Sonosite M-Turbo, FUJIFILM Sonosite Inc., Amsterdam, Netherlands), five measurements were taken post-dive. In this study, during an apical four chamber view, echocardiographic VGE signals were evaluated by frame-based bubble counting as described by Germonpré et al. (Germonpre et al., 2014). They were made at rest (without flexion) and following active provocation by two deep knee bends (with flexion (noted FLEX in the graphs)). In total, 5 videos of 15 cardiac cycles were recorded for each dive at 15 min post dive, 30, 45, 60 and 120 min. Post-dive measurements were taken after surfacing but allowing limited time for divers undressing and storing their rebreathers (CCR). They were coming back and forth to the echography station for measurements. This was made possible because divers were allocated to a group of 3 to four individuals, not entering the water at the same time and therefore not surfacing together. Every echocardiographic measurement had a duration of around 60–90 s.

At a later stage, these recordings were reviewed to analyze 10 consecutive frames in end-diastolic/protosystolic position and perform a formal bubble counting procedure. Then, the VGE peak count per heartbeat were averaged over these 10 frames, the resulting number was rounded to the closest digit and kept as the result. The counting was performed independently twice by two trained scientists acquainted with the method used (CB, CL). The numbers of VGE considered for calculation were those that reached consensus.

### 2.4 Saliva and urine measurements

Previously reported data (Arya et al., 2023) on microparticle production after a week of deep CCR diving gave us a clear view of the increasing inflammatory response during the diving week. Therefore, to evaluate the building up inflammatory process and inert gas intake in a supersaturated environment, we collected saliva, and urinary samples before and after each dive.

Saliva samples were collected by Salivette^®^ (Sarstedt, Nümbrecht, Germany), after subjects were instructed on the correct use of these devices. Approximately 1 mL of saliva was obtained and used to determine levels of Reactive Oxygen Species (ROS), and tumor necrosis factor (TNF-α).

Urine samples were collected by the participants through voluntary voiding in a sterile container. These were used to determine level of interleukin-6 (IL-6), lipid peroxidation (8-isoprostane (8-iso-PGF2)), DNA damage (8-OH-2-deoxyguanosine (8-OH-dG)) creatinine and neopterin concentrations.

Biofluids were stored at 4°C in a cooler on board and then stored in multiple aliquots at − 20°C until assayed (Giacon et al., 2022). All biological measurements were made according to the manufacturer’s instructions by previously described methods (Mrakic-Sposta et al., 2015; Mrakic-Sposta et al., 2020; Giacon et al., 2022; Bosco et al., 2023).

Reactive Oxygen Species (ROS) production rate measurement was performed by an Electron Paramagnetic Resonance (EPR) X-band spectrometer (E-Scan-Bruker^®^ BioSpin, GmbH, MA, USA). The ROS production rate was calculated from the EPR spectra saliva samples. All spectra were collected by adopting the same acquisition parameters and handled by the standards supplied by Bruker^®^ software (Win EPR System, V. 2.11). All data were, in turn, converted in absolute concentration levels (μmol·min-1).

Lipid peroxidation (8-iso-PGF2) and DNA damage (8-OH-dG) were assessed in urineby competitive immunoassay (Cayman Chemical, Ann Arbor, MI, USA) measuring concentrations in ng.mg^−1^ creatinine and pg.mg-1 creatinine, respectively. Both biomarkers’ concentrations were determined using a standard curve. Samples and standards were spectrophotometrically (Infinite M200, Tecam, Austria) read at a wavelength between 405 and 420 nm and at 412 nm, and 420 nm respectively.

TNF-α, and IL-6, saliva concentrations were determined by using ultrasensitive ELISA immunoassays kits (R&D Systems, Minneapolis, MN, USA). The assays were based on a double-antibody sandwich technique. Sample concentrations were determined spectrophotometrically for TNF-α at 412 nm, and for IL-6 at 450 nm (Infinite M200, Tecam, Austria).

The determinations were assessed in duplicate, and the inter-assay coefficient of variation was in the range indicated by the manufacturer.

Creatinine and neopterin concentrations were measured by an isocratic high-pressure liquid chromatography (HPLC) method.

### 2.5 Statistical analysis

All statistical tests were performed using a standard computer statistical package, GraphPad Prism version 9.00 for MacOS (GraphPad Software, San Diego, CA, USA).

Normality of data was verified by means of Kolmogorov-Smirnoff test allowing us to assume or not a Gaussian distribution. Since each diver is his own control, data were analyzed using one-way repeated measures ANOVA with Dunnet multiple comparison *post hoc* test. If the Gaussian distribution was not ascertained, Friedman with Dunn’s post-test was preferred.

Taking the first pre-dive measurement as 100%, biological changes were calculated at the end of each dive, allowing an appreciation of the magnitude of change rather than the absolute values. If the Gaussian distribution could be assumed, our sample mean was compared to the hypothetical mean of 100% using a One-sample *t*-test; otherwise, a Wilcoxon Signed Rank test was preferred. Then, a possible correlation was assessed through a Pearson or Spearman test and linear regression.

A threshold of *p* < 0.05 was considered statistically significant. All data are presented as mean ± standard error on the mean (SEM).

## 3 Results

There were no incidents of divers breaching their decompression profile as calculated by their dive computers. None of them developed any symptoms of DCS. All divers flew home safe and sound, more than 48 h after the last dive.

The following results refer to recordings made after the 21 man-dives performed at 100 m depth and beyond, whose basic parameters can be found in Table 3.

**TABLE 3:** Depth, Runtime, and diluent mixture composition of the analyzed dives.

	Depth (msw)	Time (min)	Oxygen (%)	Helium (%)
	102	135	6	88
	103	157	6	76
	105	156	8	73
	100	127	6	78
	102	125	6	76
	102	127	10	70
	100	84	13	59
	101	67	6	88
	102	125	6	76
	102	127	10	70
	100	84	13	59
	100	107	6	88
	122	250	9	75
	122	280	7	79
	103	116	10	70
	102	120	13	59
	122	206	8	72
	118	240	10	68
	102	190	7	81
	101	190	7	81
	121	261	8	77
Mean	106,3	155,9	8,3	74,4
SEM	1.9	13.4	0.5	1.9

The first interesting feature is that none of the measurements, VGE production or biological analysis (but for IL-6), are correlated to the exposure factor (EF) defined according to Hempleman’s formula as the product of $Depth\left(ATA\right)\times \sqrt{Total\;dive\;time\;\left(\min\right)\;}$ (Balestra et al., 2014), nor to the bubble grade when possible. Nonetheless, with a mean EF of 96 ± 6 (>25), all the analyzed dives must be considered exceptional exposure.

### 3.1 Post-dive vascular gas emboli evolution

**FIGURE 3:**
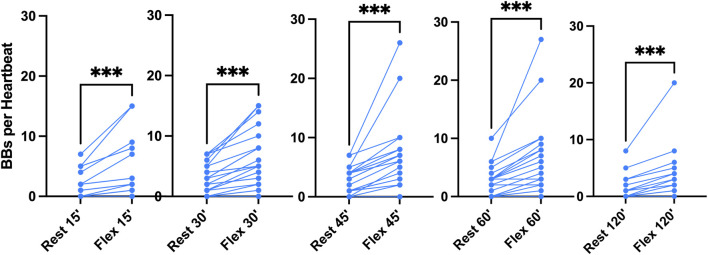
Pooled analysis of VGE Production of all 100 msw-dives (n = 21). Results are presented as pre-post graphs for every individual. The two conditions (rest and flex) are shows for every time point 15, 30, 45, 60, 120 min after diving. (Data are presented as means ± SEM; ns: not significant, ***: *p* < 0.001, ****:*p* < 0.0001; Wilcoxon matched-pair rank signed test for intra-time comparison, Friedman test for inter-time comparison).

**FIGURE 4:**
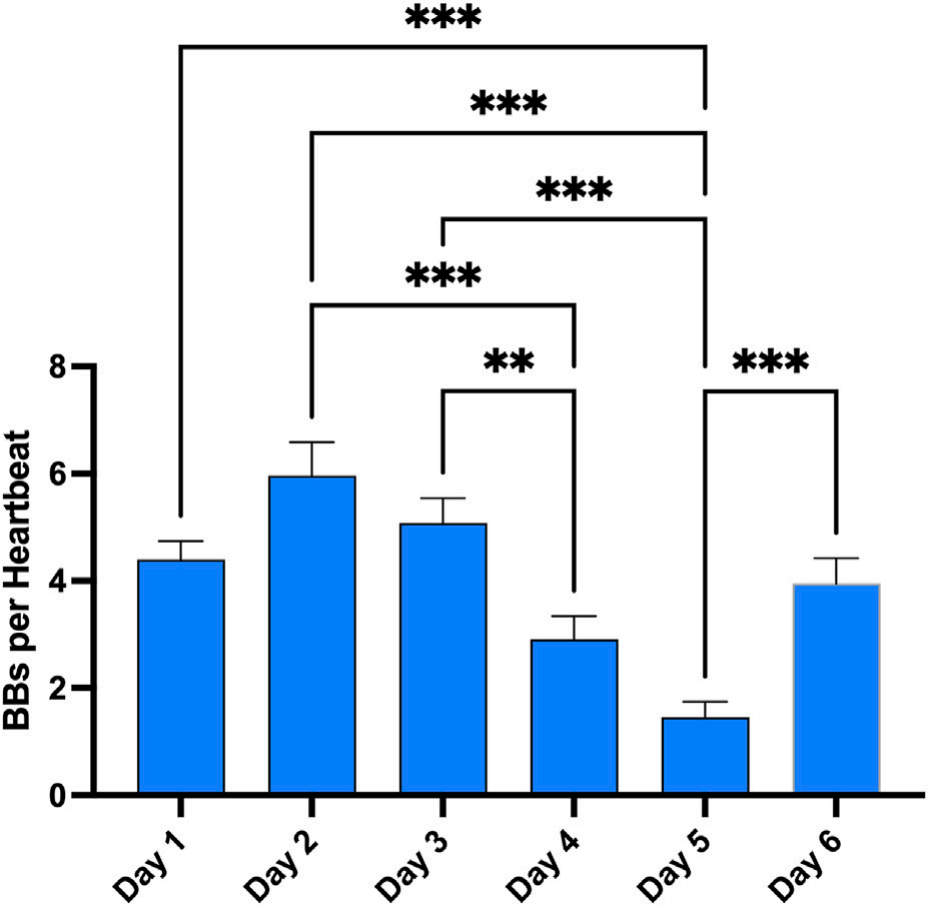
Evolution of VGE peak after the first dive of the day across the whole week. Total bubble count per heartbeat is calculated for every first dive of the day and every diver. (Data are expressed as means ± SEM; ns: not significant, **: *p* < 0.01, ***: *p* < 0.001; Friedman with Dunn’s post-test).

Results of the echocardiographic frame-based bubble counting (BBs per Heartbeat) are shown in Figures 2–4.

Given that all 100 msw-dives showed the same VGE production chronological pattern, we present the deepest dive (Maiden Wreck) as an example (Figure 2). Independently of the time of measurement, VGE production is increased by 25% ± 140% after active provocation by two deep knee bends (p between 0.01 and 0.0002, Wilcoxon matched-pair rank signed test), but for the last measurement at 120 min (*p* = 0.14, Wilcoxon matched-pair rank signed test). VGE peaked between 45 and 60 min post-dive (*p* = 0.03, Friedman test with Dunn’s post-test).

In the pooled analysis (Figure 3), it was possible to quantify the mean bubble count per cardiac cycle. At rest and after active provocation by two deep knee bends, it was respectively 2 ± 2 and 4 ± 5 at 15 min (*p* = 0.0005), 3 ± 2 and 6 ± 4 at 30 min (*p* < 0.0001), 3 ± 2 and 7 ± 6 at 45 min (*p* = 0.0001), 2 ± 3 and 6 ± 6 at 60 min (*p* = 0.0002), and 1 ± 2 and 3 ± 4 at 120 min (*p* = 0.0002). The difference between the rest and flex conditions is highly significant (Wilcoxon matched-pair rank signed test). Difference between times of measurement was highly significant both at rest (*p* = 0.0006, Friedman test) and in the flex condition (*p* = 0.0008, Friedman test). The 45-min post-dive measurement was significantly different from the other measurements in both conditions (*p* = 0.08, Dunn’s post-test).

Finally, these dives to 100 m and beyond are part of a much broader program during the whole week. Therefore, the same measurements were done after each first dive of the day to give an appreciation of the magnitude of change in VGE production across time (Figure 4). Evolution is characterized by a reduction in VGE production from day 1 to day 5, before increasing again on day 6. However, only the day 5 measurement was statistically significant compared to all other measurements (*p* < 0.0001, Friedman test with Dunn’s post-test).

### 3.2 Post-dive biological parameters

**FIGURE 5:**
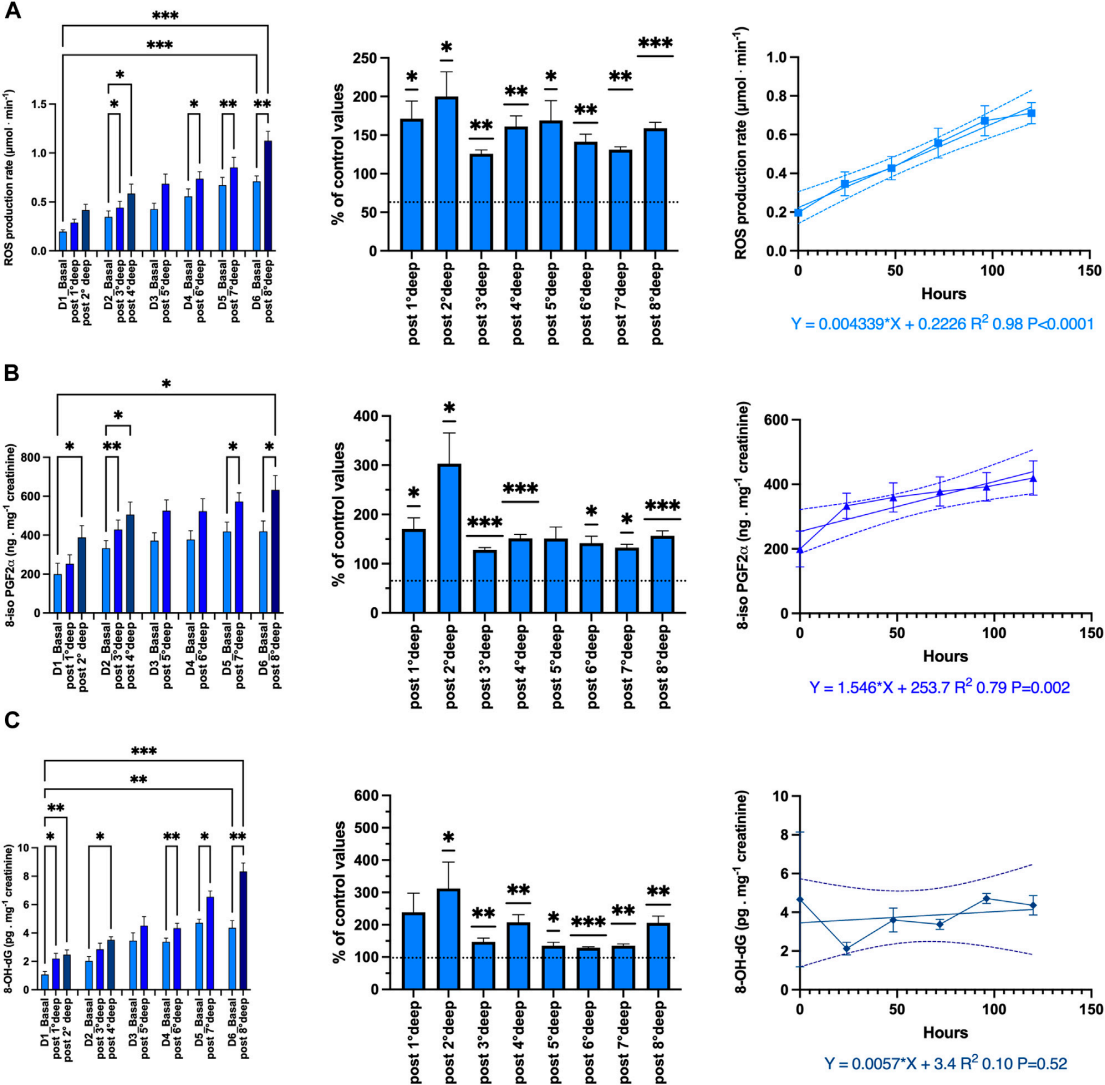
Oxidative stress: Absolute-, relative-variation and time course of: **(A)** Reactive Oxygen Species (ROS) production rate (μmol·min^−1^), **(B)** lipid peroxidation (8-iso-PGF2; ng.mg^−1^ creatinine), **(C)** DNA damage (8-OH-dG pg.mg^−1^ creatinine) before and after each dive. (Data are expressed as mean ± SEM; *p < 0.05, **p < 0.01, ***p < 0.001, one-way RM-ANOVA with Tukey’s post-test or One-sample *t*-test).

The absolute-, relative-variation and time course of the oxidative-stress-related parameters (mean ± SEM) are displayed in Figure 5.

The ROS production rate significantly increased through the diving week from 0.19 ± 0.02 to 1.13 ± 0.09 μmol.min−1 after the last dive (*p* < 0.001, one-way RM-ANOVA). When we consider the deepest dive from 89 msw and beyond, the pre- and post-dive difference is significant (one-way RM-ANOVA). With respect to the baseline values, each dive is followed by a significant mean increase of the production rate equal to 158.3% ± 6.9% (*p* = 0.021, One-sample *t*-test). However, there is no difference between each daily increase (*p* = 0.21, one-way RM-ANOVA). Finally, we observe a progressive build-up of ROS which is highly correlated to the time course (*R* ^2^ 0.98, *p* < 0.0001).

Lipid peroxidation evaluated through 8-iso-PGF2 exhibited the same pattern as ROS from 199.8 ± 55.9 to 632.7 ± 73.3 ng.mg^−1^ creatinine after the last dive (*p* < 0.0001; one-way RM-ANOVA). The increase after each dive is held constant and equal to 151.4% ± 5.7% (*p* < 0.0001, One-sample *t*-test). Correlation with time course is also significant (*R* ^2^ 0.79, *p* = 0.002).

DNA damage also exhibits a similar pattern, but for the build-up through the diving week (*R* ^2^ 0.10, *p* = 0.52). Indeed, 8-OH-dG increases from 1.07 ± 0.21 to 8.33 ± 0.6 ng.mg-1 creatinine after the last dive (*p* < 0.001; one-way RM-ANOVA). The increase after each dive is held constant at 190.4% ± 15.6% (*p* < 0.001, One-sample *t*-test). However, no progressive build-up was exhibited by DNA damage.

**FIGURE 6:**
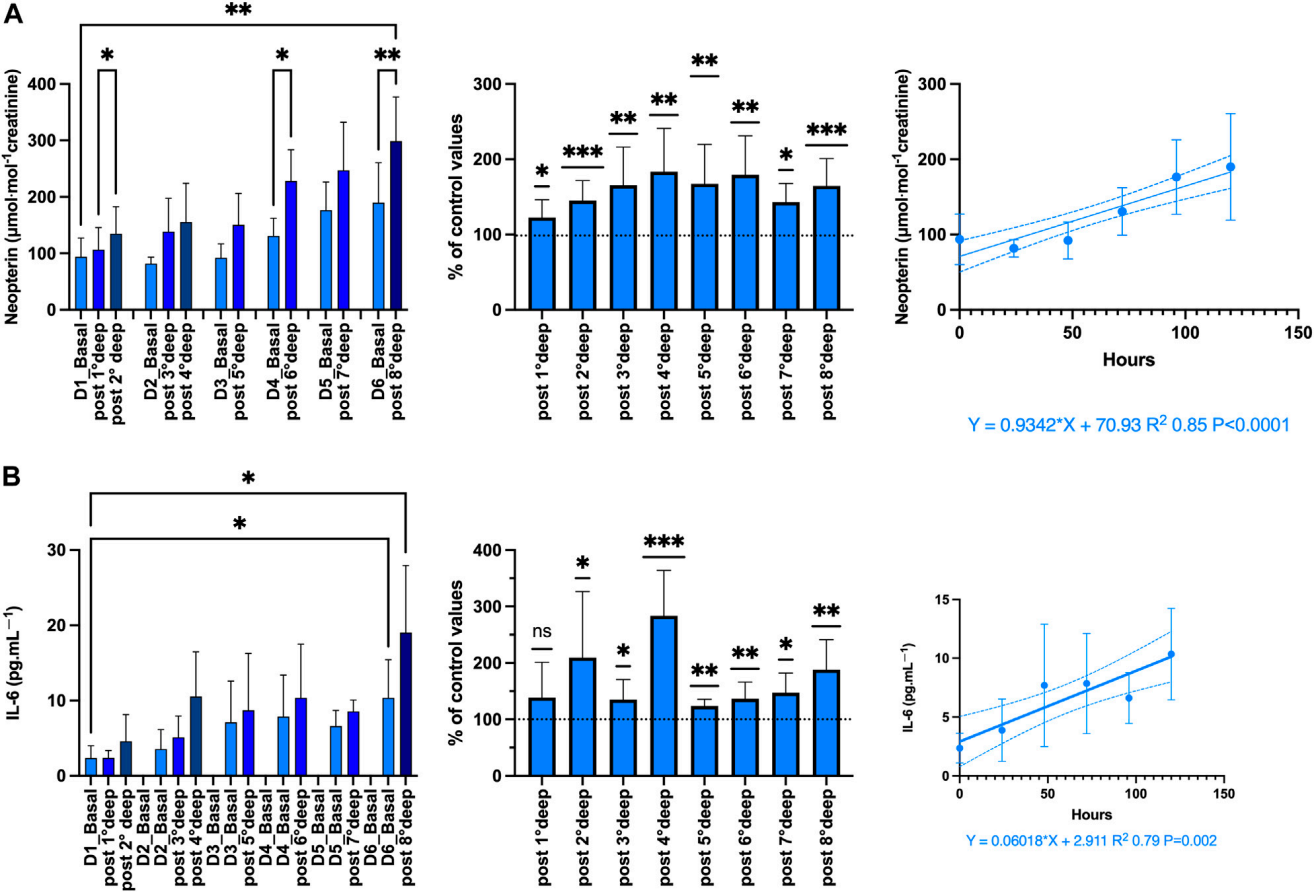
Inflammation: Absolute-, relative-variation, time course of: **(A)** Neopterin (μmol·mol−1 creatinine) **(B)** Interleukin (IL)-6 (pg.mL-1), before and after each dive. (Data are expressed as mean ± SEM; *p < 0.05, **p < 0.01, ***p < 0.001, one-way RM-ANOVA with Tukey’s post-test or One-sample *t*-test).

The inflammatory response was evaluated through the measurement of IL-6, TNF-α and neopterin. Although TNF-α plays an important role in the inflammatory response by activating the expression some proinflammatory genes and the leukocyte adhesion to vessels (Jarosz-Griffiths et al., 2019), no significant changes were observed in the present study. Indeed, with respect to the baseline values, each dive is followed by a slight variation of 101.4% ± 0.3% (*p* = 0.07, One-sample *t*-test) from 37.1 ± 2.9 to 41.4 ± 3.4 pg.mL-1 (*p* = 0.42; one-way RM-ANOVA). Therefore, only IL-6 and neopterin are shown in Figure 6.

The proinflammatory state is confirmed by both the increase of neopterin from 93.7 ± 11.2 to 299 ± 25.9 μmol·mol^−1^ creatinine (*p* = 0.005; one-way RM-ANOVA) and IL-6 from 2.35 ± 0.54 to 19.5 ± 2.96 pg.mL-1 (*p* < 0.001; one-way RM-ANOVA) after the last dive. The variation after each dive was held constant around 160.1% ± 5.6% and 176.3% ± 11.9% respectively (*p* < 0.001 and *p* < 0.0001 respectively, One-sample *t*-test). These variations were responsible for a progressive linear build-up strongly correlated with time-course (*R* ^2^ 0.85, *p* < 0.0001 and *R* ^2^ 0.79, *p* = 0.002, respectively).

## 4 Discussion

Although the exact sequence of events leading to DCS is not fully elucidated yet, it is acknowledged that DCS is a multifactorial pathology. Indeed, it involves several physiological pathways such as inflammation (Thom et al., 2011), coagulation (Lambrechts et al., 2018) or vascular dysfunction (Mazur et al., 2016) that unite to produce what is called decompression stress. Nowadays, research projects use VGE data as a surrogate endpoint to evaluate decompression stress (Doolette, 2016; Balestra et al., 2019). Although VGE detection techniques improve (from acoustic Doppler to visual 2D echocardiography to second harmonics echocardiography), there is obviously a size limit below which no VGE will be detected (Papadopoulou et al., 2013; Papadopoulou et al., 2014). The limits of the sensibility of VGE detection methods imply that small, undetected VGE [less than 22 μm (Hills and Butler, 1981)] might pass the pulmonary vasculature [which acts as a bubble “filter” (Carturan et al., 1999)] and exert influence in the arterial vascular bed. Therefore, the limitations associated with VGE grade as a surrogate indicator of decompression stress may explain some possible misinterpretation.

According to our results, the VGE production pattern over time suggests a biphasic mechanism. Indeed, VGE start forming during the off-gassing of tissues in the decompression (ascent) phase of a dive and are believed to result from the triggering of bubble precursors (nuclei) into growth. The precise mechanism of micronuclei formation is still debated, with possible sites being located on facilitating endothelial surface regions with surfactants, hydrophobic surfaces, or crevices (Papadopoulou et al., 2013; Papadopoulou et al., 2014; Arieli et al., 2015; Papadopoulou et al., 2015) (see Figure 1). However, the presumed micronuclei-originated VGE production is consistent with the observation that a certain form of “acclimatization” to decompression stress seems to exist, with a higher probability of VGE for the first dives after a period of non-diving (Zanchi et al., 2014; Arieli et al., 2015; Balestra et al., 2022b). This is consistent with the progressive reduction of VGE production (depletion of pre-existing micronuclei) even in the presence of provocative dives to 100 msw and beyond.

However, previous studies have also pointed out significant inter-subject variability to VGE for the same diving exposure (Papadopoulou et al., 2018). There is also a large intra-individual variation, indicating that diving time and nitrogen pressure are not the only determinants of VGE formation. Indeed, oxidative stress is not necessarily a manifestation of toxicity and organ damage, but it seems to be involved in the pro-inflammatory response to diving among humans.

Normally, in response to tissue injury, non-immune and immune cells released cytokines such as IL-1β, TNF-α or interferon-g (IFN-γ), which in turns are important for the expression of inflammatory mediators such as prostaglandins, leukotrienes, platelet-activating factor or IL-6 (Hirano, 2021). However, TNF-α was not modified in the present study. Nonetheless, based on lipid peroxidation, and IL-6, our results suggest this proinflammatory response is well present, requiring alternatives hypotheses.

Firstly, studies with humans have demonstrated that during the high-pressure phase and before decompression, there are elevations in microparticles (MPs) number and those expressing filamentous (F-) actin on the membrane surface (Brett et al., 2019; Bhopale et al., 2021). Similar responses occur in a murine DCS model. Although their pathophysiological role in diving remains unclear, if MPs are purified and injected into naïve mice, they cause a similar collection of symptoms as seen in decompressed mice (Thom et al., 2011; Thom et al., 2018; Bhopale et al., 2021). Finally, it has also to be reminded that a previous study demonstrated that some individuals may exhibit a more exuberant NOS2 or IL-1β production, such that nucleation-site-carrying MPs generate more VGE in response to diving (Arya et al., 2023).

Secondly, with a PpO_2_ setpoint between 1.3 and 1.6 ATA, exposure to constant hyperoxia cannot be separated from CCR diving, which may have led to those significant increases. Indeed, hyperbaric hyperoxia has been associated with an increase in oxidative stress leading to nuclear factor (erythroid-derived)-like 2 (NRF2) and nuclear factor-kappa B NF-κB activation, accompanied by the synthesis of glutathione (GSH) (Fratantonio et al., 2021). These pathways have already been considered in scuba diving (Theunissen et al., 2013; Cialoni et al., 2019) and seem to be mediated through the expression of nitric oxide (NO), whose enzymes are at the forefront of the ROS signaling system (Burtenshaw et al., 2017). Indeed, NO has a potent biphasic effect on NF-κB activity and possesses the ability to both up- and downregulate the expression of a number of proinflammatory proteins, including IL-6 (Connelly et al., 2001). Also, maintaining IL-6 levels in a physiological range is necessary to keep inflammatory responses under control (Stoner et al., 2013). Since, the expression of IL-6 is mainly regulated by the activation of NF-κB (Brasier, 2010), which is dependent on oxygen levels and ROS production, we can hypothesize that the oxy-inflammation is at the system’s core of decompression (Brizzolari et al., 2023; Mrakic-Sposta et al., 2023; Vezzoli et al., 2024). This hypothesis is also supported by the 8-iso-PGF2 increase, which depends on the availability of hydroxyl radicals and by neopterin measurements. This is consistent with former studies demonstrating that 8-iso- PGF2 is increased during hyperoxic oral breathing (Levenez et al., 2022), which was the case during this study. Neopterin is synthesized by human IFN-γ-stimulated macrophages and is indicative of a proinflammatory status, paralleling the increase of ROS which depends on PpO_2_. Although controversial, it has been proposed that NF-κB may also play a role in interleukin-10 (IL-10) regulation through interactions with distal enhancers at the IL-10 locus (Saraiva et al., 2005). This might be of importance. Indeed, concomitantly with the systemic inflammatory response syndrome (SIRS), the body develops a compensatory anti-inflammatory response syndrome (CARS) in response to injury. CARS is a complex pattern of immunologic responses that depends on the production of anti-inflammatory mediators, such as IL-10 (Ward et al., 2008). The difference is that while SIRS is tasked with limiting the extent of injury, CARS is aimed at dampening the inflammatory reaction, allowing restoration of homeostasis. However, once triggered, CARS, if prolonged, may also be detrimental to the host. An imbalance between the two signals leaves inflammation unchecked, resulting in further cellular and tissue damage. Much research now suggests that the timing and relative magnitude of this response have a profound impact on patient outcomes (Iyer and Cheng, 2012). When diving is involved, to the best of our knowledge, only one study has addressed IL-10 without significant results (Arya et al., 2023). However, this was done after a single exposure. Since helium exposure elicits cardiac preconditioning by decreasing expression of the proinflammatory markers CD11b and ICAM-1 on leukocytes and attenuating the expression of the procoagulant markers CD42b and PSGL-1 on platelets (Lucchinetti et al., 2009), it is then a reasonable assumption that repetitive exposure to both hyperoxia and helium might modify IL-10 expression and/or the SIRS/CARS ratio as a result of those deep dives. Finally, it is interesting to note that despite a constant daily variation, we observe a progressive build-up of ROS and 8-iso-PGF2 while this is not the case for DNA damage. This suggests that the complex endogenous antioxidant system, including enzymes such as superoxide dismutase, GSH and catalase is exceeded (Leveque et al., 2023). Therefore, we can assume that these different mechanisms may combine themselves and when oxy-inflammation reaches a certain level, it exceeds hormetic coping mechanisms, which is also suggested by the strong correlation between the duration of exposure in days and inflammatory markers (ROS, lipid peroxidation, Neopterin and IL-6).

This study is a field observation and has some limitations for which further investigation is needed. The main limitation of the present study is, obviously, the relatively small number of participants. Moreover, the participants were not homogenous or necessarily similar in body composition (age, weight, fat/lean mass distribution), number of dives performed in total during the study, and were not tested to be “consistent bubblers” before the experiment [this would have required at least three extra identical “control” dives (Germonpré and Balestra, 2017)]. Therefore, an appropriately powered study could have produced results that could have been different. Nonetheless, it would have been difficult to either recruit more participants or analyze a larger group in a timely manner. However, based on the magnitude of the variation and the level of significance, we can assume some objective relevance. Despite the limitations, this study builds on established modern methods of evaluation of decompression stress and current theories of VGE generation. The measured effects are consistent with the theoretical rationale and do not require complicated new hypotheses. Finally, the equipment used for these experiments is readily available, inviting other research groups to repeat the study.

## 5 Conclusion

This study emphasizes the role of oxy-inflammation that could explain the latter increase in VGE production after the deepest dive at the end of the week. Indeed, above a certain level of inflammation that can be reached along the repetition of dives, some hormetic controlling mechanisms are exceeded, and the ensuing MP’s serve as second-generation micronuclei increasing the risk of decompression illness. Since this study demonstrated that all modifications were not related to the exposure, it must be hypothesized that decompression risk is related to the capacity to cope with acute phase reactants. Although difficult to perform due to the provocative aspect of deep diving, larger studies are encouraged to confirm these results.

## Data Availability

The raw data supporting the conclusion of this article will be made available by the authors, without undue reservation.
